# 6-Bromo-1-(1,2-propadien­yl)-3-(2-propyn­yl)-1*H*-imidazo[4,5-*b*]pyridin-2(3*H*)-one

**DOI:** 10.1107/S1600536810007695

**Published:** 2010-03-06

**Authors:** S. Dahmani, A. Haoudi, F. Capet, El Mokhtar Essassi, Seik Weng Ng

**Affiliations:** a Laboratoire de Chimie Organique Appliquée, Faculté des Sciences et Techniques, Université Sidi Mohamed Ben Abdallah, Fés, Morocco; b Unité de Catalyse et de Chimie du Solide, Ecole Nationale Supérieure de Chimie de Lille, Lille, France; c Laboratoire de Chimie Organique Hétérocyclique, Pôle de Compétences Pharmacochimie, Université Mohammed V-Agdal, BP 1014 Avenue Ibn Batout, Rabat, Morocco; d Department of Chemistry, University of Malaya, 50603 Kuala Lumpur, Malaysia

## Abstract

The reaction of propargyl bromide and 6-bromo-1,3-dihydro­imidazo[4,5-*b*]pyridin-2-one in refluxing dimethyl­formamide yields the title compound, C_12_H_8_BrN_3_O, which features nitro­gen-bound propadienyl and propynyl substituents. The imidazolopyridine fused ring is planar (r.m.s. deviation = 0.012 Å); the propadienyl chain is coplanar with the fused ring as it is conjugated with it, whereas the propynyl chain is not as the nitro­gen-bound C atom is a methyl­ene linkage. The acetyl­enic H atom is hydrogen bonded to the carbonyl O atom of an adjacent mol­ecule, forming a helical chain runnning along the *b* axis.

## Related literature

For the crystal structures of other imidazo[4,5-*b*]pyridin-2-ones, see: Kourafalos *et al.* (2002 ▶); Meanwell *et al.* (1995 ▶).
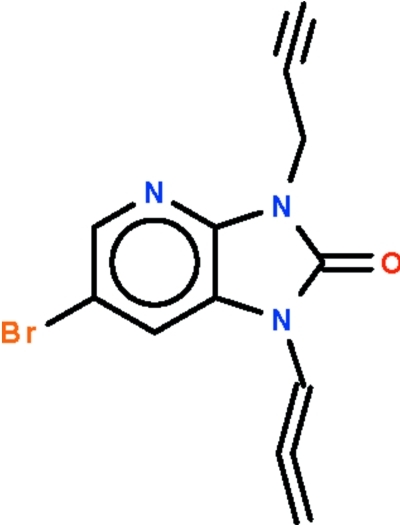


## Experimental

### 

#### Crystal data

C_12_H_8_BrN_3_O
*M* *_r_* = 290.12
Monoclinic, 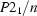

*a* = 9.6369 (4) Å
*b* = 9.3086 (4) Å
*c* = 13.5481 (5) Å
β = 99.123 (2)°
*V* = 1199.97 (8) Å^3^
*Z* = 4
Mo *K*α radiation
μ = 3.41 mm^−1^
*T* = 293 K
0.55 × 0.35 × 0.30 mm

#### Data collection

Bruker X8 APEXII diffractometer
Absorption correction: multi-scan (*SADABS*; Sheldrick, 1996 ▶) *T*_min_ = 0.256, *T*_max_ = 0.428
51411 measured reflections
3393 independent reflections
2577 reflections with *I* > 2σ(*I*)
*R*_int_ = 0.040

#### Refinement

*R*[*F* ^2^ > 2σ(*F* ^2^)] = 0.031
*wR*(*F* ^2^) = 0.087
*S* = 1.02
3393 reflections
166 parameters
3 restraints
H atoms treated by a mixture of independent and constrained refinement
Δρ_max_ = 0.49 e Å^−3^
Δρ_min_ = −0.63 e Å^−3^

### 

Data collection: *APEX2* (Bruker, 2008 ▶); cell refinement: *SAINT* (Bruker, 2008 ▶); data reduction: *SAINT*; program(s) used to solve structure: *SHELXS97* (Sheldrick, 2008 ▶); program(s) used to refine structure: *SHELXL97* (Sheldrick, 2008 ▶); molecular graphics: *X-SEED* (Barbour, 2001 ▶); software used to prepare material for publication: *publCIF* (Westrip, 2010 ▶).

## Supplementary Material

Crystal structure: contains datablocks global, I. DOI: 10.1107/S1600536810007695/hg2649sup1.cif

Structure factors: contains datablocks I. DOI: 10.1107/S1600536810007695/hg2649Isup2.hkl

Additional supplementary materials:  crystallographic information; 3D view; checkCIF report

## Figures and Tables

**Table 1:** Hydrogen-bond geometry (Å, °)

*D*—H⋯*A*	*D*—H	H⋯*A*	*D*⋯*A*	*D*—H⋯*A*
C12—H12⋯O1^i^	0.94 (1)	2.22 (1)	3.161 (3)	173 (2)

Symmetry code: (i) 
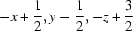
.

**Fig. 1.**
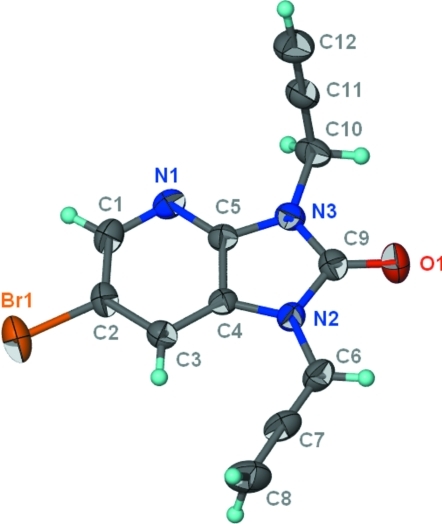
Thermal ellipsoid plot (Barbour, 2001) of C12H8BrN3O at the 50% probability level; hydrogen atoms are drawn as spheres of arbitrary radius.

## supplementary crystallographic information

### Experimental

To a solution of 6-bromo-1,3-dihydro-imidazo[4,5-*b*]pyridin-2-one (1 mmol), potassium carbonate (4 mmol) and tetra-*n*-butylammonium bromide (0.1 mmol) in DMF (20 ml) was added propargyl bromide (2.5 mmol). The solution was refluxed for 48 hours. After completion of the reaction (as monitored byTLC), the salt was filtered and the solvent was removed under reduced pressure. The residue was purified by column chromatography on silica gel by using an ethyl acetate/hexane (1/1) mixture as eluent. Slow evaporation of the solvent furnished colorless crystals.

### Refinement

Carbon-bound H-atoms were placed in calculated positions (C—H 0.93 to 0.97 Å) and were included in the refinement in the riding model approximation, with *U*(H) set to 1.2*U*(C). The terminal acetylenic and allenic H-atoms were located in a difference Fourier map, and were refined with a distance restraint of C–H 0.95±0.01 Å; their temperature factors were refined.

### Crystal data

**Table:**

C_12_H_8_BrN_3_O	*F*(000) = 576
*M**_r_* = 290.12	*D*_x_ = 1.606 Mg m^−^^3^
Monoclinic, *P*2_1_/*n*	Mo *K*α radiation, λ = 0.71073 Å
Hall symbol: -P 2yn	Cell parameters from 9975 reflections
*a* = 9.6369 (4) Å	θ = 2.4–25.6°
*b* = 9.3086 (4) Å	µ = 3.41 mm^−^^1^
*c* = 13.5481 (5) Å	*T* = 293 K
β = 99.123 (2)°	Irregular, colorless
*V* = 1199.97 (8) Å^3^	0.55 × 0.35 × 0.30 mm
*Z* = 4	

### Data collection

**Table:**

Bruker X8 APEXII diffractometer	3393 independent reflections
Radiation source: fine-focus sealed tube	2577 reflections with *I* > 2σ(*I*)
graphite	*R*_int_ = 0.040
φ and ω scans	θ_max_ = 29.7°, θ_min_ = 2.7°
Absorption correction: multi-scan (*SADABS*; Sheldrick, 1996)	*h* = −13→13
*T*_min_ = 0.256, *T*_max_ = 0.428	*k* = −12→12
51411 measured reflections	*l* = −18→18

### Refinement

**Table:**

Refinement on *F*^2^	Primary atom site location: structure-invariant direct methods
Least-squares matrix: full	Secondary atom site location: difference Fourier map
*R*[*F*^2^ > 2σ(*F*^2^)] = 0.031	Hydrogen site location: inferred from neighbouring sites
*wR*(*F*^2^) = 0.087	H atoms treated by a mixture of independent and constrained refinement
*S* = 1.02	*w* = 1/[σ^2^(*F*_o_^2^) + (0.0413*P*)^2^ + 0.4635*P*] where *P* = (*F*_o_^2^ + 2*F*_c_^2^)/3
3393 reflections	(Δ/σ)_max_ = 0.001
166 parameters	Δρ_max_ = 0.49 e Å^−^^3^
3 restraints	Δρ_min_ = −0.63 e Å^−^^3^

### Fractional atomic coordinates and isotropic or equivalent isotropic displacement parameters (Å^2^)

**Table:**

	*x*	*y*	*z*	*U*_iso_*/*U*_eq_	
Br1	0.40648 (3)	0.39876 (3)	0.231050 (17)	0.06785 (11)	
O1	0.49655 (17)	0.95432 (17)	0.64205 (11)	0.0577 (4)	
N1	0.21863 (16)	0.64109 (19)	0.42594 (13)	0.0453 (4)	
N2	0.54742 (15)	0.78896 (16)	0.52363 (11)	0.0395 (3)	
N3	0.32605 (16)	0.81244 (17)	0.54674 (11)	0.0417 (3)	
C1	0.2477 (2)	0.5495 (2)	0.35479 (15)	0.0490 (4)	
H1	0.1744	0.4972	0.3188	0.059*	
C2	0.3815 (2)	0.53039 (19)	0.33326 (13)	0.0435 (4)	
C3	0.4981 (2)	0.60309 (19)	0.38454 (13)	0.0399 (4)	
H3	0.5887	0.5895	0.3708	0.048*	
C4	0.46792 (17)	0.69616 (18)	0.45684 (12)	0.0346 (3)	
C5	0.32815 (18)	0.71031 (18)	0.47294 (12)	0.0365 (3)	
C6	0.6938 (2)	0.8150 (2)	0.53701 (16)	0.0503 (5)	
H6	0.7302	0.8828	0.5845	0.060*	
C7	0.7806 (2)	0.7510 (2)	0.48776 (17)	0.0529 (5)	
C8	0.8709 (3)	0.6872 (4)	0.4413 (2)	0.0750 (8)	
H81	0.916 (3)	0.602 (2)	0.467 (3)	0.106 (12)*	
H82	0.890 (3)	0.722 (3)	0.3788 (13)	0.089 (10)*	
C9	0.4605 (2)	0.8628 (2)	0.57909 (14)	0.0416 (4)	
C10	0.2020 (2)	0.8584 (2)	0.58740 (16)	0.0509 (5)	
H10A	0.1252	0.8736	0.5328	0.061*	
H10B	0.2218	0.9494	0.6217	0.061*	
C11	0.1586 (2)	0.7538 (2)	0.65707 (15)	0.0521 (5)	
C12	0.1208 (3)	0.6715 (3)	0.71186 (19)	0.0694 (7)	
H12	0.093 (3)	0.601 (2)	0.7550 (18)	0.088 (11)*	

### Atomic displacement parameters (Å^2^)

**Table:**

	*U*^11^	*U*^22^	*U*^33^	*U*^12^	*U*^13^	*U*^23^
Br1	0.0918 (2)	0.05653 (15)	0.05116 (14)	0.01376 (12)	−0.00107 (12)	−0.01710 (9)
O1	0.0645 (9)	0.0499 (8)	0.0563 (9)	0.0031 (7)	0.0024 (7)	−0.0158 (7)
N1	0.0357 (8)	0.0481 (9)	0.0513 (9)	−0.0027 (7)	0.0046 (7)	0.0022 (7)
N2	0.0367 (7)	0.0396 (8)	0.0417 (7)	−0.0023 (6)	0.0050 (6)	−0.0021 (6)
N3	0.0417 (8)	0.0425 (8)	0.0431 (8)	0.0011 (6)	0.0133 (6)	−0.0014 (6)
C1	0.0469 (10)	0.0461 (10)	0.0504 (10)	−0.0050 (8)	−0.0035 (8)	−0.0017 (8)
C2	0.0555 (11)	0.0369 (9)	0.0362 (8)	0.0034 (8)	0.0016 (7)	−0.0014 (7)
C3	0.0408 (9)	0.0411 (9)	0.0385 (8)	0.0031 (7)	0.0085 (7)	0.0024 (7)
C4	0.0351 (8)	0.0350 (8)	0.0334 (7)	−0.0008 (6)	0.0051 (6)	0.0060 (6)
C5	0.0386 (8)	0.0350 (8)	0.0364 (8)	0.0006 (7)	0.0079 (6)	0.0057 (6)
C6	0.0381 (9)	0.0515 (11)	0.0592 (12)	−0.0078 (8)	0.0015 (8)	−0.0032 (9)
C7	0.0343 (9)	0.0597 (12)	0.0628 (12)	−0.0064 (9)	0.0017 (9)	0.0098 (10)
C8	0.0396 (11)	0.101 (2)	0.0867 (19)	0.0039 (13)	0.0176 (12)	0.0060 (17)
C9	0.0474 (10)	0.0370 (9)	0.0402 (9)	0.0029 (7)	0.0061 (7)	0.0014 (7)
C10	0.0512 (11)	0.0491 (11)	0.0570 (12)	0.0097 (9)	0.0224 (9)	0.0029 (9)
C11	0.0510 (11)	0.0614 (12)	0.0461 (10)	0.0007 (9)	0.0149 (8)	−0.0033 (9)
C12	0.0748 (16)	0.0808 (18)	0.0555 (13)	−0.0120 (14)	0.0197 (12)	0.0087 (12)

### Geometric parameters (Å, °)

**Table:**

Br1—C2	1.8928 (18)	C3—C4	1.373 (2)
O1—C9	1.216 (2)	C3—H3	0.9300
N1—C5	1.312 (2)	C4—C5	1.404 (2)
N1—C1	1.349 (3)	C6—C7	1.295 (3)
N2—C4	1.390 (2)	C6—H6	0.9300
N2—C9	1.393 (2)	C7—C8	1.296 (4)
N2—C6	1.414 (2)	C8—H81	0.95 (1)
N3—C5	1.382 (2)	C8—H82	0.95 (1)
N3—C9	1.382 (2)	C10—C11	1.463 (3)
N3—C10	1.458 (2)	C10—H10A	0.9700
C1—C2	1.378 (3)	C10—H10B	0.9700
C1—H1	0.9300	C11—C12	1.164 (3)
C2—C3	1.398 (3)	C12—H12	0.94 (1)
			
C5—N1—C1	114.50 (16)	N1—C5—C4	126.59 (17)
C4—N2—C9	109.96 (14)	N3—C5—C4	107.53 (15)
C4—N2—C6	128.72 (16)	C7—C6—N2	124.62 (19)
C9—N2—C6	121.29 (16)	C7—C6—H6	117.7
C5—N3—C9	110.03 (15)	N2—C6—H6	117.7
C5—N3—C10	125.63 (16)	C8—C7—C6	178.0 (3)
C9—N3—C10	124.33 (16)	C7—C8—H81	121 (2)
N1—C1—C2	122.72 (18)	C7—C8—H82	120.7 (19)
N1—C1—H1	118.6	H81—C8—H82	118 (3)
C2—C1—H1	118.6	O1—C9—N3	127.55 (18)
C1—C2—C3	122.49 (17)	O1—C9—N2	126.48 (18)
C1—C2—Br1	118.07 (14)	N3—C9—N2	105.97 (15)
C3—C2—Br1	119.43 (14)	N3—C10—C11	112.56 (17)
C4—C3—C2	114.56 (16)	N3—C10—H10A	109.1
C4—C3—H3	122.7	C11—C10—H10A	109.1
C2—C3—H3	122.7	N3—C10—H10B	109.1
C3—C4—N2	134.39 (16)	C11—C10—H10B	109.1
C3—C4—C5	119.12 (16)	H10A—C10—H10B	107.8
N2—C4—C5	106.49 (15)	C12—C11—C10	178.4 (3)
N1—C5—N3	125.87 (16)	C11—C12—H12	177.2 (19)
			
C5—N1—C1—C2	0.0 (3)	C3—C4—C5—N1	0.9 (3)
N1—C1—C2—C3	0.9 (3)	N2—C4—C5—N1	−179.58 (17)
N1—C1—C2—Br1	−179.76 (15)	C3—C4—C5—N3	−178.29 (15)
C1—C2—C3—C4	−0.8 (3)	N2—C4—C5—N3	1.26 (18)
Br1—C2—C3—C4	179.81 (12)	C4—N2—C6—C7	−1.4 (3)
C2—C3—C4—N2	−179.36 (18)	C9—N2—C6—C7	−179.6 (2)
C2—C3—C4—C5	0.0 (2)	C5—N3—C9—O1	178.90 (19)
C9—N2—C4—C3	178.04 (18)	C10—N3—C9—O1	−2.7 (3)
C6—N2—C4—C3	−0.3 (3)	C5—N3—C9—N2	−0.2 (2)
C9—N2—C4—C5	−1.41 (19)	C10—N3—C9—N2	178.17 (16)
C6—N2—C4—C5	−179.75 (17)	C4—N2—C9—O1	−178.10 (19)
C1—N1—C5—N3	178.16 (17)	C6—N2—C9—O1	0.4 (3)
C1—N1—C5—C4	−0.9 (3)	C4—N2—C9—N3	1.00 (19)
C9—N3—C5—N1	−179.84 (17)	C6—N2—C9—N3	179.49 (16)
C10—N3—C5—N1	1.8 (3)	C5—N3—C10—C11	76.4 (2)
C9—N3—C5—C4	−0.67 (19)	C9—N3—C10—C11	−101.7 (2)
C10—N3—C5—C4	−179.00 (16)		

### Hydrogen-bond geometry (Å, °)

**Table:**

*D*—H···*A*	*D*—H	H···*A*	*D*···*A*	*D*—H···*A*
C12—H12···O1^i^	0.94 (1)	2.22 (1)	3.161 (3)	173 (2)

Symmetry codes: (i) −*x*+1/2, *y*−1/2, −*z*+3/2.
